# Piezo1‐Mediated Mechanotransduction: Orchestrating the Dynamic Response of Podocytes and Parietal Epithelial Cells to Mechanical Stress

**DOI:** 10.1111/apha.70173

**Published:** 2026-02-08

**Authors:** Maria Elena Melica, Giulia Antonelli, Anna Julie Peired, Laura Lasagni

**Affiliations:** ^1^ Nephrology and Dialysis Unit Meyer Children's Hospital IRCCS Florence Italy; ^2^ Department of Experimental and Clinical Biomedical Sciences “Mario Serio” University of Florence Florence Italy

**Keywords:** mechanobiology, Piezo1, podocyte, podocyte progenitors

## Abstract

**Aim:**

The glomerulus is a specialized microvascular unit that filters plasma through the coordinated function of podocytes and parietal epithelial cells (PECs). From this perspective, the glomerulus functions like a living hydrogeological filtration system. This review aims to integrate mechanobiology and hydrogeology, reframing podocytes and PECs as active regulators in a pressure‐driven network, with Piezo1 central to glomerular homeostasis, adaptation, and pathology.

**Methods:**

This review integrates existing literature on glomerular biology, mechanosensitive signaling, and epithelial cell function, focusing on podocytes, PECs, and mechanosensitive structures such as the Piezo1 channel.

**Results:**

Podocytes form interdigitating foot processes connected by the slit diaphragm, forming both a selective barrier against protein loss and a mechanosensory interface. Through mechanosensitive structures, such as the Piezo1 channel, podocytes detect variations in hydrostatic pressure and transduce these cues into intracellular signaling that regulates permeability and preserves structural integrity. Sustained mechanical stress, however, can compromise podocyte function and viability. PECs line Bowman capsule, forming an impermeable boundary surrounding the filtration core. Once considered passive, PECs exhibit dynamic properties: some retain progenitor‐like potential, contributing to repair, whereas others promote fibrosis in disease conditions. In this analogy, blood flow replaces groundwater while the multilayered filtration barrier mirrors stratified geological formations. Podocytes function as biological piezometers—sensing pressure and modulating filtration—while PECs resemble aquicludes, defining impermeable boundaries that can constrain or reshape the system under mechanical or inflammatory challenges.

**Conclusion:**

By integrating mechanobiology and hydrogeology, this review reframes the glomerulus as a living, pressure‐driven filtration system in which podocytes and PECs act as active regulators rather than passive structural elements, with Piezo1 playing a central role in glomerular homeostasis, adaptation, and pathology.

## 1 Introduction: The Glomerulus as a Living Multi‐Layered (Hydrogeological) System

**FIGURE 1:**
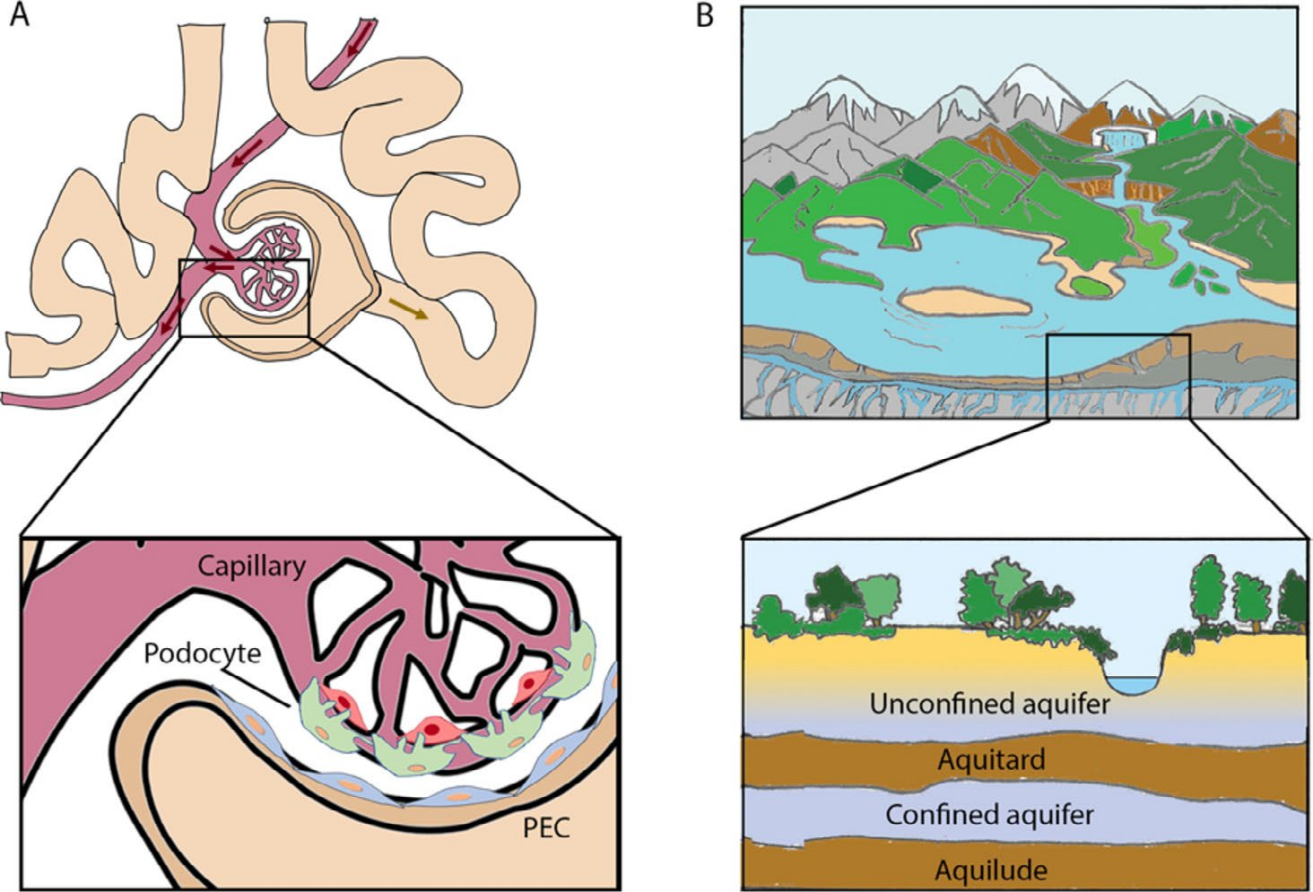
The glomerulus as a living multi‐layered hydrogeological system. (A) Schematic of the glomerular structure. The capillary with blood flow (red) resembles the unconfined aquifer; the glomerular filtration barrier composed of endothelial cells, the glomerular basement membrane and podocytes, resembles the aquitard; the Bowman space, which collects the glomerular filtrate (red arrows) or preurine (yellow arrow), resembles the confined aquifer; and the Bowman capsule lined with PECs resembles the aquiclude. (B) Schematic of a hydrogeological system. The unconfined aquifer lacks impermeable barrier above it, while the confined aquifer is overlain by an aquitard, a bed of low permeability, and underlain by an aquiclude, a solid and impermeable region.

The renal glomerulus is a highly specialized microvascular structure that functions as the primary filtration unit of the kidney. It consists of a tuft of capillaries lined by fenestrated endothelial cells, the glomerular basement membrane (GBM), and differentiated podocytes, which together form the glomerular filtration barrier (Figure 1A) [1]. Podocytes play a pivotal role in maintaining the selectivity and integrity of the filtration barrier through interdigitating foot processes and slit diaphragm complexes, which regulate size‐ and charge‐selective permeability [2, 3]. The Bowman capsule, composed of a basal membrane and the parietal epithelial cells (PECs), forms a simple squamous, nonfiltering layer that maintains the capsule's integrity not allowing fluid or protein egress (Figure 1A) [4].

The process of glomerular ultrafiltration is governed by the balance of Starling forces acting across the capillary wall [5]. Specifically, glomerular capillary hydrostatic pressure drives fluid into Bowman space, whereas capillary oncotic pressure (from plasma proteins) and Bowman capsule hydrostatic pressure oppose filtration [6]. The fluid in Bowman capsule is virtually protein‐free, and therefore its oncotic pressure does not significantly influence the glomerular filtration process. However, the presence of proteins in Bowman space, which can occur under pathological conditions, may alter this pressure and affect the filtration dynamics [6]. Thus, the glomerular filtration rate (the volume of plasma filtered by glomeruli per unit of time, GFR) is highly sensitive to glomerular pressure [6]. By lining Bowman capsule and shaping Bowman space, PECs define the counteracting hydrostatic pressure, and through paracrine signaling and phenotypic plasticity, they influence podocyte survival and barrier function [7, 8]. Under stress or injury, PECs can either contribute to adaptive repair or maladaptive scarring, directly impacting the balance of forces and the efficiency of ultrafiltration [7, 8].

To sense and respond to mechanical stress modulating barrier function under changing hemodynamic conditions, podocytes possess specific mechanosensors: (i) the actin cytoskeleton and focal adhesion complexes dynamically transmit and adapt to tension [9]. (ii) Mechanosensitive channels are essential for translating glomerular filtration pressure and stretch into biochemical signals. TRPC6 is the most prominent sensor at the slit diaphragm, where both gain‐of‐function and loss‐of‐function mutations are linked to focal segmental glomerulosclerosis (FSGS) [10]. This channel works in a fine‐tuned balance with TRPC5, a closely related mechanosensor; while TRPC6 promotes stress fiber formation via RhoA, TRPC5 activation during mechanical stress triggers Rac1‐mediated actin remodeling, leading to podocyte effacement [11, 12]. Additionally, TRPV4 acts as a critical mechanoreceptor that modulates calcium influx and cytoskeletal organization in response to cellular swelling and stretch [13]. To prevent calcium overload, BKCa channels (large‐conductance (Ca^2+^)‐activated (K^+^) channels) provide a protective feedback mechanism by hyperpolarizing the membrane and stabilizing the actin‐regulating protein synaptopodin [14]. Finally, Piezo1 converts mechanical stimuli into calcium signals and modulates cytoskeletal organization and slit diaphragm integrity [15]. (iii) Integrins provide bidirectional signaling with the GBM, coupling extracellular matrix stiffness to intracellular signaling pathways [16].

The dynamic interactions between cellular structures, extracellular matrices, and physical forces governing filtration and homeostasis in the glomerulus, bear resemblance to geological processes, in which the Earth's crust continuously senses and adapts to the pressure and flow of underlying fluids [17, 18]. Just as in the groundwater systems, the aquifer consists of porous rock, sand or sediment saturated with water, acting as a natural reservoir and conduit for groundwater flow (Figure 1B), in the glomerulus, the filtration barrier—composed of fenestrated endothelial cells, the GBM, and podocyte foot processes—resembles a “living” aquifer that permits selective fluid flow (Figure 1A,B). In groundwater systems, piezometers measure pressure; similarly, in the glomerulus, podocytes equipped with Piezo1 channels and other mechanosensors serve as biological piezometers, sensing and responding to mechanical signals. Aquicludes‐impermeable layers that regulate fluid containment and flow‐find their biological parallel in the PECs lining the Bowman capsule, which maintain the structural integrity of the filtration space and help modulate pressure dynamics.

Although necessarily imperfect, this metaphor highlights how the glomerular tuft behaves like a confined groundwater reservoir where fluid enters under pressure, and specialized cells gauge and restrict that flow. Such an analogy may be useful because both biological and hydrogeological systems are governed by common physical principles—pressure gradients, hydraulic permeability, and resistance to flow—that dictate the distribution and movement of fluids within a constrained environment (Figure 1A,B). By framing glomerular filtration in terms familiar to hydrogeology, this perspective underscores the universality of these physical laws and may facilitate conceptual models that integrate biomechanics, fluid dynamics, and structural adaptation.

## 2 Hydromechanical Coupling in Glomerular (and Geological) Systems

**FIGURE 2:**
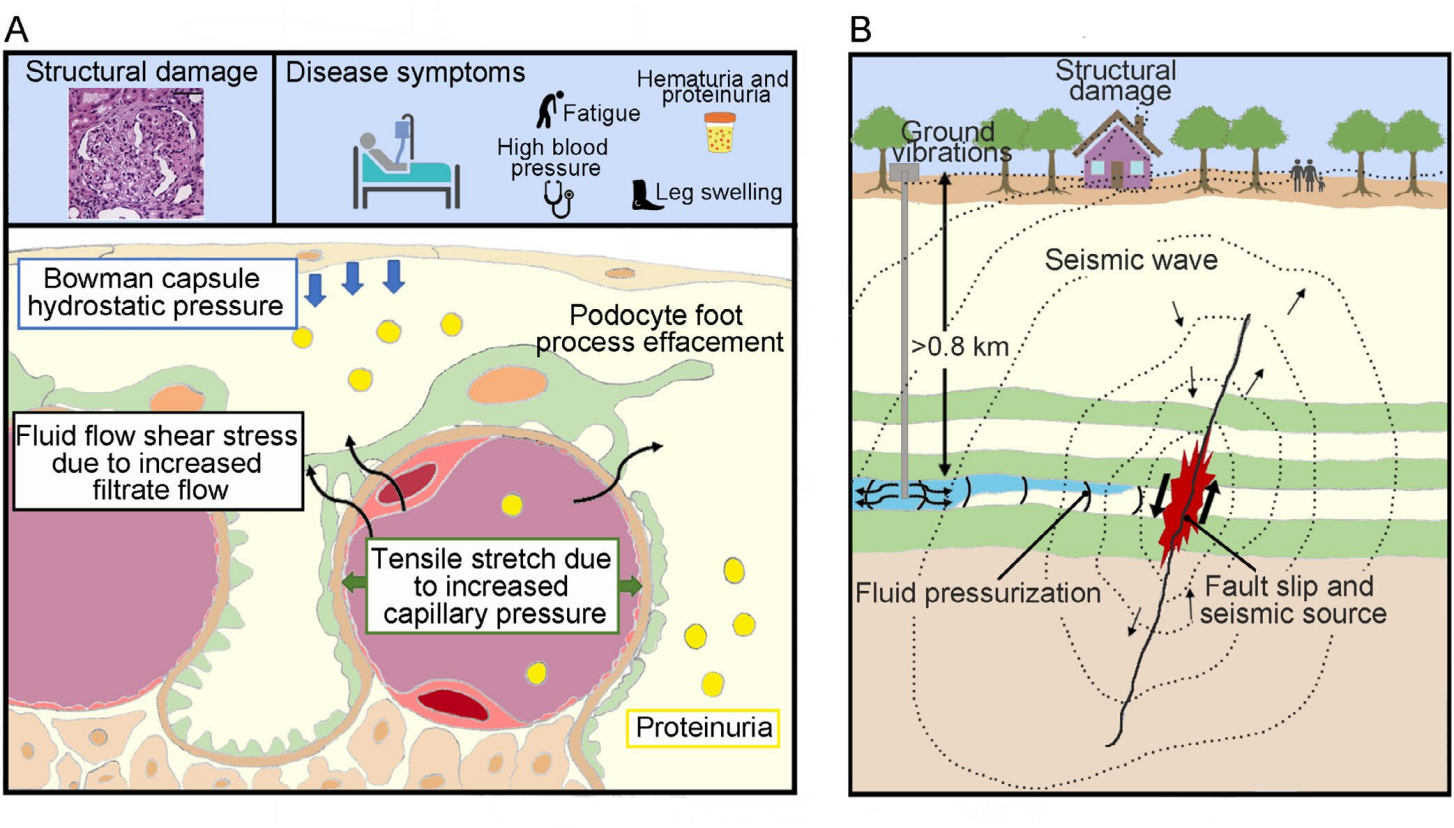
Hydromechanical coupling in glomerular and hydrogeological systems. (A) Schematic of mechanical forces acting on the glomerular filtration barrier. Changes in circumferential tensile stretch from capillary expansion and fluid shear stress from filtrate flow can induce structural glomerular damage and the development of glomerular disease manifestations such as proteinuria. (B) Changes in hydromechanical coupling by injection or removal of fluids in aquifer can induce fault reactivation and earthquakes, potentially affecting surface infrastructures and human populations.

At the heart of the glomerular filtration systems lies hydromechanical coupling: the interaction between fluid forces and structural elements. In the kidney, this coupling allows the glomerulus to balance effective blood filtration with preservation of barrier integrity and homeostasis. Normal autoregulatory mechanisms (e.g., afferent arteriole myogenic responses) buffer large fluctuations, but pathological increases in intraglomerular pressure (as in hypertension or diabetes) raise transmural wall tension and filtration. Notably, elevated glomerular capillary pressure and hyperfiltration cause podocyte stress and can induce glomerulosclerosis (Figure 2A) [15]. In addition to hydrostatic load, flowing fluid exerts shear stress on glomerular cells. First, as blood flows through the capillaries, endothelial cells sense flow‐dependent shear [19]. Moreover, the ultrafiltrate streaming through the narrow podocyte slits and into Bowman space imparts a fluid‐shear force directly on podocyte foot processes, especially relevant in pathologic situations when it contributes to the loss of viable podocytes (Figure 2A) [20]. Thus, disruptions in hydromechanical coupling can lead to structural glomerular alterations, dysfunction, and finally to kidney disease (Figure 2A).

Evaluations of physical forces in vivo in glomeruli have been hampered by a lack of quantitative functional data. Thus, the calculations of the magnitude of mechanical forces produced by filtrate flow through filtration slits have been derived from multiscale simulation models and mathematical estimations [18, 21, 22, 23, 24]. Fuhrmann et al. recently applied computational fluid dynamics to estimate the shear stress challenge to the filtration barrier during glomerular filtration in rats and produced a high‐resolution spatial model of the filtration process [20]. Using this numerical flow simulation, they suggested that the filtration barrier experiences high levels of shear and pressure stress, estimating a mean wall shear stress of 39 Pa on podocyte plasma membranes with a maximum wall shear stress of 152 Pa on podocyte plasma membranes and 250 Pa on the internal slit diaphragm surface [20]. These values exceed previous estimates by 13‐ to 50‐fold higher [23], and are substantially higher than estimates for shear forces on vascular endothelium [25]. By performing extensive parameter variations, such as reducing the filtration slit width, increasing filtrate flow velocity, and altering the viscous resistance of the slit diaphragm, the authors concluded that the filtration barrier experiences high levels of shear and pressure stress that account for the detachment of injured podocytes from the GBM—a hallmark in many glomerular diseases [20], proved by the detection of both viable and dead podocytes in patient's urine [26, 27, 28, 29, 30]. Thus, local hemodynamic effects and local stresses on cells, together with hemodynamic changes, may contribute to podocyte detachment and subsequent glomerular sclerosis, as demonstrated by elegant studies that built on classic observations, and add state‐of‐the‐art imaging, modeling, intravital microscopy, and ultrastructural geometry analyses [21, 31]. These studies provide new insights into potential mechanisms underlying podocyte injury and loss, and strengthen the importance of studies focused on the mechanobiology of podocytes [32].

As in the glomerulus, theoretical approaches and numerical tools are available in hydrogeological systems to analyze the forces regulating hydromechanical coupling. Alterations in these forces—triggered by fluid injection, groundwater extraction, or natural/mechanical stress—can lead to significant geological and societal consequences. When groundwater is excessively extracted, the reduction in pore pressure within the aquifer can cause the surrounding geological formations to compact, resulting in land subsidence at the surface which could lead to damage to infrastructure such as buildings, roads, and pipelines, as well as increased flood risk in low‐lying areas (Figure 2B). Conversely, fluid injection, such as in geothermal energy production or oil and gas operations, can increase pore pressure within the aquifer or surrounding formations. This can cause surface uplift and may also alter the stress state in the subsurface, sometimes triggering induced seismicity that poses risks to nearby communities and critical infrastructure [33, 34] (Figure 2B).

## 3 Podocytes as Living PIEZOmeters

**FIGURE 3:**
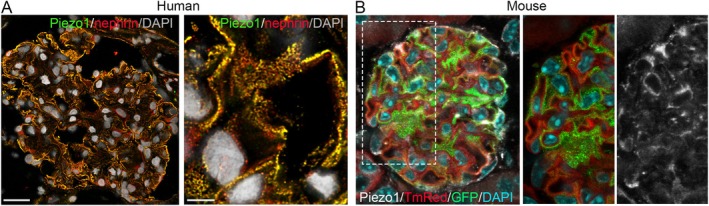
Piezo1 expression in human and mouse podocytes. (A) Immunofluorescence of a human glomerulus showing Piezo1 (green) colocalized with the podocyte marker nephrin (NPHS1, red). Piezo1 localizes to foot processes (arrows), consistent with its role as a mechanosensor. (B) Immunofluorescence of a mouse glomerulus showing Piezo1 (white) expression in podocytes, in which GFP (green) is expressed under the control of NPHS2 promoter (modified from Melica et al. JASN 2025 [42], article distributed under the terms of the Creative Commons Attribution‐Non Commercial‐No Derivatives License 4.0 (CCBY‐NC‐ND)).

Inside the glomerulus, podocytes act as highly mechanosensitive cells that detect and transduce biomechanical forces via mechanosensors like TRPC6, P2X4, and cytoskeleton‐associated proteins, initiating downstream signaling cascades that regulate cytoskeletal dynamics, cell survival, and barrier function [15, 35, 36, 37]. The recent discovery of the evolutionarily conserved PIEZO channel family, including PIEZO1 and PIEZO2 in mammals, as bona fide mechanically activated cation channels, has added a new component to the long list of mechanosensitive ion channels expressed by different cell types [38]. Structurally, Piezo1 forms a trimeric, propeller‐like complex that opens a central pore in response to mechanical stimuli, allowing the influx of cations, primarily calcium. Calcium entry triggers downstream signaling pathways that regulate cellular responses to mechanical stress, including changes in cytoskeletal organization, gene expression, and cell survival. Piezo1 thus acts as a crucial sensor that converts physical forces into biochemical signals, enabling cells to adapt to dynamic mechanical environments [38]. The expression and the role of Piezo1 in podocyte have been recently conducted across various kidney diseases, including hypertensive nephropathy (HTN) [39], lupus nephritis (LN) [40], diabetic kidney disease (DKD) [41], and Adriamycin nephropathy (ADN) [42] using proteomic analysis, single‐cell RNA sequencing, and imaging techniques [39, 42, 43, 44, 45]. Proteomic analysis of isolated mouse podocytes showed a ~12% enrichment of Piezo1 compared to other glomerular cells [44]. Using RNAscope in situ hybridization and immunofluorescence staining, researchers observed Piezo1 expression in podocytes—notably along foot processes and in cell bodies, both in human and in mouse, as clearly shown by Melica et al. (Figure 3A,B) [39, 42, 43, 44, 45]. These findings have been corroborated by single‐nucleus RNA‐sequencing from human kidney tissue [44], and single‐cell RNA sequencing from human and mouse glomeruli [40, 42, 45]. In the mouse glomeruli dataset, Piezo1 expression was evident in ~40% of the isolated podocytes and associated with enrichment of the “Rho matrix” and the “Signaling through Rho‐GTPases” gene sets [42]. Moreover, these data established that not all podocytes express the Piezo1 channel in healthy conditions, and that its expression increased in diseases [39, 40, 41, 42].

The first study of the role of Piezo1 in vivo has been performed by Ogino et al. in a mouse model of HTN [39]. By RNAscope imaging, the authors reported Piezo1 upregulation in podocytes in the HTN mice, accompanied by the induction of podocyte injury‐related markers. However, specific knockout (KO) of the Piezo1 gene in vivo in podocytes to confirm its involvement in HTN was lacking. Recently, the same research group generated podocyte‐specific Piezo1 KO mice and demonstrated that they did not spontaneously develop podocyte injury but developed severe podocyte injury with overt proteinuria under hypertensive conditions, surprisingly suggesting a protective role of Piezo1 [46].

**FIGURE 4:**
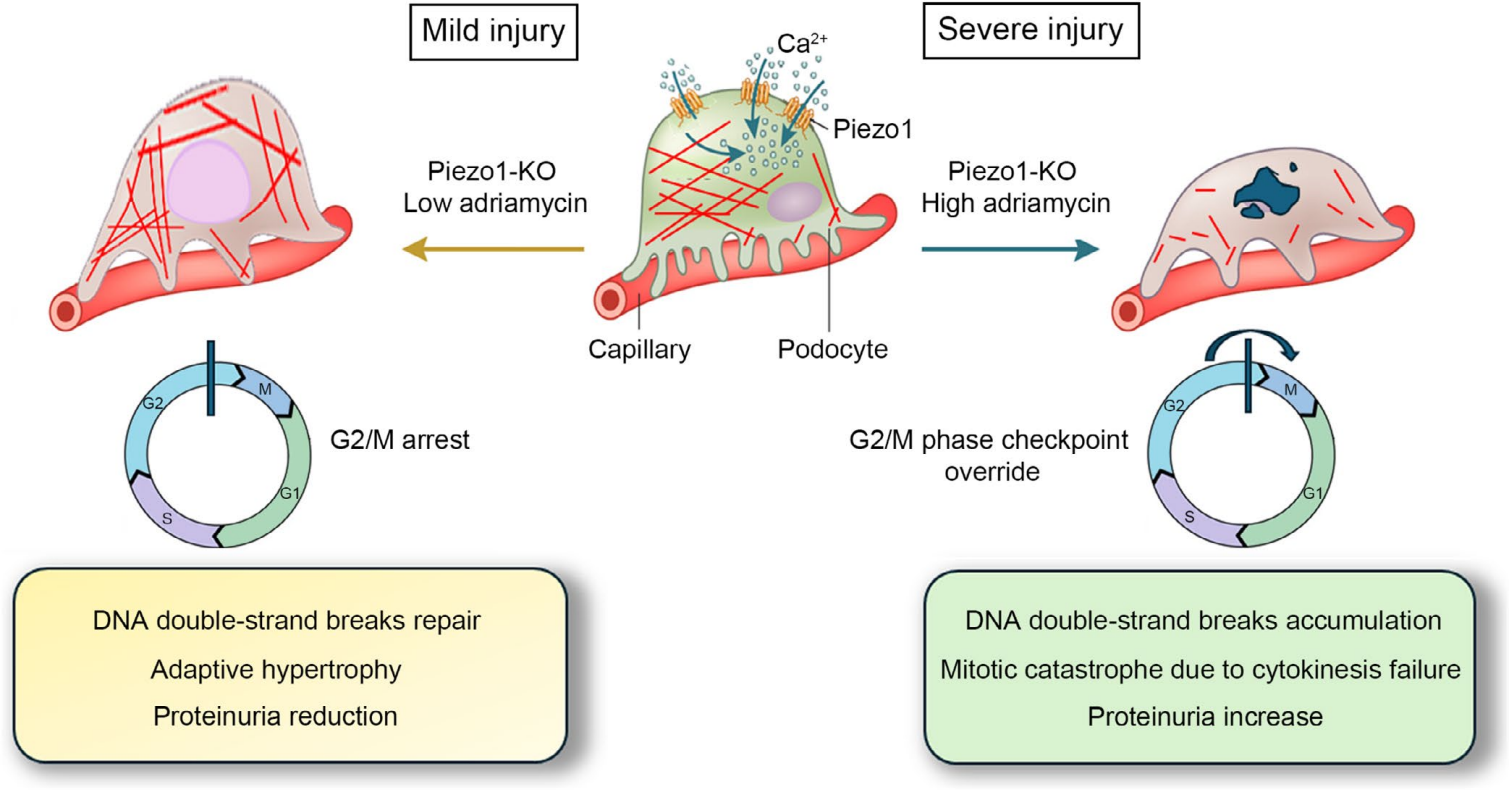
Dual role of Piezo1 knockout (KO) in podocytes depending on injury severity. In conditions of mild injury (left panel), Piezo1 KO podocytes reenter the cell cycle but arrest at the G2/M checkpoint. During the G2/M checkpoint arrest, cells repair DNA double‐strand breaks induced by Adriamycin and undergo hypertrophy. All these events exert a protective effect. In contrast, under severe injury (right panel), podocytes reenter the cell cycle but override the G2/M checkpoint, DNA double‐strand breaks accumulate, leading to mitotic catastrophe, podocyte loss, and worsening of proteinuria. This dual effect highlights how the impact of Piezo1 deletion is critically dependent on the severity of podocyte damage and the subsequent cell cycle response.

Contrasting results on the protective versus detrimental role of Piezo1 in podocytes have been reported also in other kidney disease models. Fu et al. investigated the contribution of Piezo1 to podocyte injury and proteinuria progression in the pristane‐induced LN model in mice with podocyte‐specific Piezo1 KO driven by the NPHS1 promoter (Podo‐Piezo1−/− mice) [40]. Podocyte‐specific Piezo1 deletion induced protective effects on the progression of proteinuria and foot process effacement in the murine LN model, but did not lead to a significant decrease in the levels of serum creatinine and blood urea nitrogen [40]. The same authors reported beneficial effects of Piezo1 deletion in two other mouse models of kidney injury. Indeed, in the anti‐glomerular basement membrane glomerulonephritis model, following Piezo1‐KO in podocytes, they observed a reduction in the percentage of glomeruli with crescents, attenuated proteinuria, mitigated podocyte foot process effacement, and preserved podocyte number compared with their Podo‐Piezo1+/+ counterparts. Finally, in the ADR mouse model, Podo‐Piezo1−/− mice also showed improvement of glomerular lesions, accompanied by decreased proteinuria and greater podocyte number [40]. More recently, Melica et al. reported contrasting results in the ADR model in mice with podocyte‐specific Piezo1 KO driven by the NPHS2 promoter [42], with the NPHS2‐Piezo1‐KO mice exhibiting higher proteinuria and worsened glomerular injury starting from day 14 post‐ADR induction. Using optical clearing of kidney tissue, immunofluorescent staining for nephrin, and 3D reconstruction, the authors observed reduced foot process coverage, in association with foot process effacement as well as a thickening of the GBM in the NPHS2‐Piezo1‐KO mice, thus supporting, with morphological data, the functional deterioration of the glomeruli [42]. A careful analysis of the two studies to identify differences explaining the conflicting results could reveal crucial aspects of Piezo1's role in podocytes. Aside from the use of different promoters to drive podocyte‐specific KO—which might contribute to the discrepancies, although the extent of its impact remains unclear—the main difference between the two studies lies in the severity of podocyte damage induced in the mice. Both studies were performed in the C57BL/6 mouse strain. In this strain, described as resistant to ADR, no or only minimal histologic and ultrastructural changes have been reported when animals received a standard dose of Adriamycin [47, 48, 49] as in the work by Fu et al. [40] On the contrary, Melica et al. induced the nephropathy by two successive retro‐orbital injections of Adriamycin [42]. The difference in podocyte damage severity induced is huge as demonstrated by the albumin/creatinine ratio measured at Day 28. Thus, the opposite results observed would suggest that the Piezo1 channel mediates different effects depending on the severity of the damage, being its KO beneficial when the damage is mild and detrimental when the damage is severe. A potential explanation for the divergent effects of Piezo1 deletion in mild versus severe podocyte injury can be drawn from studies on epithelial cell biology showing that Piezo1 activation promotes proliferation in response to mechanical stretch, and studies exploring the essential role of Piezo1 during cytokinesis [50, 51, 52, 53]. In the context of epithelial homeostasis, Piezo1 activation exhibits context‐dependent, seemingly opposite effects on cell behavior, including cell proliferation vs. apoptosis, cell extrusion vs. retention, or protection vs. injury. In epithelial cells, mechanical cues sensed via Piezo1 channels trigger calcium influx, leading to ERK1/2 phosphorylation and subsequent transcription of cyclin B1, allowing G2‐arrested cells to enter mitosis. Accordingly, Piezo1 inhibition blocks stretch‐induced proliferation by preventing cyclin B synthesis [52]. Podocytes are terminally differentiated epithelial cells that reside in G0/G1 phase and do not normally proliferate [54]. However, in response to pathological stimuli—such as glomerular hypertension or toxin‐induced damage—they may aberrantly reenter the cell cycle. Due to their complex cytoskeletal architecture and loss of mitotic capacity, podocytes typically arrest at the G2/M checkpoint. This arrest can trigger two alternative fates depending on the extent of damage and the cell's ability to adapt: adaptive hypertrophy or mitotic catastrophe [55, 56]. Hypertrophy may represent a compensatory mechanism (so‐called “optimal hypertrophy”) to maintain glomerular filtration in the face of podocyte loss [57, 58]. In mild injury settings, in the absence of Piezo1 activity, G2 arrested podocytes did not enter mitosis and remain hypertrophied arrested at the G2/M phase checkpoint, during which the low level of DNA double strand breaks induced by low amounts of Adriamycin are corrected [59]. In this state, they are more resistant to stressing conditions than the Piezo1 expressing podocytes. In contrast, under severe injury, podocytes are pushed to override the G2/M checkpoint. The consequences are the incomplete repair of DNA double strand breaks, acquisition of a multinucleated phenotype due to the absence of Piezo1 at cytokinesis [53] and precipitated mitotic catastrophe [56, 57, 60]. Indeed, Piezo1 plays a critical role in cytokinesis by sensing mechanical forces generated during cell division. During the late stages of mitosis, tension at the cleavage furrow activates Piezo1, allowing localized calcium influx. This calcium signal regulates actomyosin contractility and coordinates the constriction of the contractile ring, ensuring proper ingression of the cleavage furrow and successful separation of daughter cells. Dysregulation of Piezo1 activity can lead to cytokinesis failure, resulting in multinucleated cells or mitotic catastrophe, highlighting its essential function in mechanically coordinating cell division [53]. Thus, the outcome of Piezo1 modulation may depend critically on the injury context, the extent of cellular stress, and the podocytes' ability to undergo compensatory hypertrophy versus mitotic failure (Figure 4).

The complexity of the biological scenario is further underscored by findings from a study conducted in Drosophila nephrocytes, the podocyte homolog in the fly [44]. In this cellular context, both Piezo1 depletion and activation/overexpression have been reported to preserve podocyte morphology and function. Loss‐of‐function analysis in Piezo‐depleted nephrocytes revealed a severe morphological and functional phenotype, but elevated Piezo expression levels also resulted in a severe nephrocyte phenotype [44]. All this evidence outlines a “Janus‐face” for Piezo1 with both the level of Piezo1 expression/activity and the severity of podocyte damage impacting on the podocytopathy outcome, either directly or through the activation of still largely unexplored compensatory mechanisms. A “Janus” face for Piezo1 has already been described in the cardiovascular system [61]; in endothelial cells, when the expression level of Piezo1 is within the safe range of its threshold it can play a protective role in atherosclerosis; under turbulent flow however, the expression level of Piezo1 exceeds the threshold which causes vascular inflammation reaction and brings adverse effects on cardiovascular health [18].

The complex role of Piezo1 likely lies in the type of intracellular message it activates, namely the Ca^2+^ flow. Ca^2+^ is a ubiquitous, complex, multifactorial, and dynamic intracellular signal critical to many cellular processes [62]. The Ca^2+^ signaling mechanism is extremely versatile both in terms of speed, amplitude, and spatio‐temporal patterning. Thus, varying outcomes of calcium‐dependent processes may result from differences in the duration and amplitude of the calcium signal, as well as the potential influence of other external stimuli acting synergistically on the podocytes.

Further contributing to the complexity of Piezo1 regulation is the identification of Piezo1 isoforms, such as Piezo1.1 [63]. This isoform is broadly expressed in both mouse and human cell lines as well as in primary tissues, and can form mechanosensitive cation channels either as homomers or as heteromers with Piezo1. Notably, Piezo1.1 exhibits enhanced mechanosensitivity and higher single‐channel conductance, suggesting it may function as a more responsive mechanotransducer compared to canonical Piezo1 [63]. The distinct calcium permeability and downstream signaling profiles of Piezo1 and Piezo1.1 suggest a potential mechanism by which cells can precisely regulate intracellular Ca^2+^ dynamics [63]. The ability of Piezo1.1 to differ in mechanosensitivity and single‐channel conductance, and to assemble with canonical Piezo1 subunits into trimeric channels, raises the possibility that mechanotransduction thresholds can be finely modulated through differential expression of Piezo isoforms. In the kidney, this mechanism may explain how podocytes exhibit distinct mechanosensory behaviors in response to hemodynamic stress. Studying the physiological function of the Piezo1.1 splice variant in glomerular cells prompts several key questions: Which Piezo1 isoforms are present in podocytes? Do they assemble into heteromeric channels in vivo? Is Piezo1 splicing regulated developmentally, influenced by shear stress, or altered in kidney disease? How does isoform variation affect force transmission during filtration? Elucidating the regulatory mechanisms that govern Piezo isoform expression in the kidney may provide new insights into the cellular basis of renal mechanotransduction under physiological and pathological conditions. Interestingly, recent findings provide compelling evidence that alternative splicing plays a critical role in enabling podocytes to adapt to mechanical stress by regulating actin dynamics. In particular, Mattias and colleagues showed that applying stretch to podocytes induces two distinct Shroom3 splice variants [64]. One variant is uniquely expressed in podocytes and, when knocked down, leads to severe F‐actin disruption [64]. The other variant appears to facilitate cytoskeletal remodeling in response to pressure alterations, demonstrating functional specialization in podocyte mechanoadaptation [64].

In vitro studies have been used to better clarify the mechanisms by which Piezo1 mediates its effects in podocytes. Piezo1 activation induced by the specific agonist Yoda1 in conditionally immortalized rat podocyte cell line activated Rac1, a member of the Rho family of small G‐proteins, and induced the expression of gene markers associated with podocyte injury [39]. Yoda1 stimulation in human and murine immortalized podocytes induced cytoskeleton remodeling characterized by a reduction in stress fiber, the appearance of actin‐rich lamellopodia, upregulation of phospho‐paxillin, and formation of focal adhesion [40]. As a consequence, directional movement of cells was inhibited, an effect that was reverted by addition of GsMTx4 or silencing of Piezo1 [40]. The authors reported, moreover, that Piezo1 expression was upregulated by inflammatory cytokines (IL‐6, TNF‐α and IFN‐γ), soluble urokinase Plasminogen Activator Receptor, and by its activation [40]. In the manuscript by Weiwei et al., podocytes were cultured on polyacrylamide hydrogels with a modulus of 4–35 kPa, representing the transition from normal to fibrotic stiffness in the kidney cortex [41]. The protein abundance of Piezo1 increased in immortalized human podocyte cells cultured on hydrogels with stiffness of 8, 20, and 35 kPa, with the highest abundance observed at 20 kPa. Moreover, flow cytometry with a calcium probe showed that intracellular calcium flow increased with increasing stiffness, with the most pronounced effect observed at 20 kPa [41]. Additionally, the apoptosis assay revealed significantly higher podocyte apoptotic rates in the 20 and 35 kPa stiffness ranges. Mechanistically, Piezo1 activation triggered a signaling loop involving NFATc1 and TRPC6, leading to increased calcium influx, perpetuating podocyte injury [41].

Melica et al. performed in vitro experiments using human podocytes obtained from the differentiation of podocyte progenitors through 48 h treatment with all‐trans‐retinoic acid [42]. Silencing of Piezo1 with Locked Nucleic Acid (LNA) gapmer antisense oligonucleotide (LNA‐gapmer) did not alter the differentiation process but induced alteration in F‐actin organization, accumulation of double‐strand DNA breaks, followed by mitotic catastrophe and higher susceptibility to death in culture conditions that reproduce the shear stress associated with hyperfiltration. Exposure of Piezo1‐silenced podocytes to a FAK activator or the Rho Activator II reduced the apoptosis induced by shear stress, suggesting that in these conditions Piezo1 signaling maintains podocyte survival by regulating the integrin‐signaling through FAK and the RhoA activity [42]. Interestingly, when Piezo1 signaling through RhoA signaling is predominant, such as in the study by Melica et al. and Mikami et al., Piezo1 seems protective, whereas it appears pathogenic when acting by Rac1 signaling, as reported by Fu et al. and Li et al. [40, 41, 42, 46].

In conclusion, podocytes are increasingly recognized as highly specialized mechanosensors that gauge hydrostatic and shear forces within the glomerular tuft. By converting these mechanical cues into biochemical signals, podocytes regulate cytoskeletal dynamics, slit diaphragm integrity, and glomerular permeability, thereby providing continuous “feedback” on the mechanical state of the filtration barrier. In this sense, they can be conceptualized as biological counterparts of piezometers—devices used to measure groundwater pressure at specific depths—which yield essential information on hydraulic gradients, flow direction, and aquifer stability [17, 18, 34]. Just as piezometers translate subsurface hydraulic conditions into actionable data for understanding fluid dynamics in geological systems, podocytes sense and transduce hemodynamic forces into adaptive or maladaptive responses that shape renal function. This analogy underscores the potential value of framing podocytes as active pressure sensors, capable of providing real‐time information on glomerular homeostasis and stress, and highlights their dual role in maintaining barrier selectivity under physiological conditions and triggering pathological remodeling when adaptive thresholds are exceeded.

Although preclinical studies using Piezo1 agonists, such as Yoda1, or inhibitors like GsMTx4, have demonstrated the channel's critical role in podocyte mechanotransduction, cytoskeletal dynamics, and survival, highlighting its therapeutic potential, the clinical translation of Piezo1‐targeted therapies for podocytopathies could face significant challenges. Key hurdles include limited selectivity of available modulators, the heterogeneity of podocyte injury across different disease contexts, constraints in effective drug delivery to the glomerulus, and the lack of predictive or pharmacodynamic biomarkers. Overcoming these translational barriers will require the development of highly selective Piezo1 modulators, tailored treatment strategies guided by mechanobiology‐based biomarkers, and optimized delivery systems to target podocytes specifically.

## 4 Parietal Epithelial Cells: A Biological Aquiclude

PECs constitute the simple squamous epithelial lining the Bowman capsule [1]. They form a continuous impermeable sheet wrapping the glomerular tuft, meeting the proximal tubule cells at the urinary pole and blending into the podocytes at the vascular pole [1]. Unlike podocytes, PECs are not part of the filtration barrier; instead, they serve a structural role supporting the rigid scaffold of the capsule and helping confine filtrate under pressure by maintaining the integrity and low‐compliance wall of the Bowman capsule. However, their mechanical role in intraglomerular pressure handling remains underappreciated. Distension of the Bowman capsule due to increased preurine flux in hyperfiltration alters intraglomerular pressure dynamics and raises tensile stress on the parietal epithelial layer. Moreover, shear stress increases due to the higher velocity of ultrafiltrate flow [65]. Thus, PEC geometry and adhesion likely contribute to glomerular homeostasis, possibly providing back pressure and impacting transversal shear stress on podocytes in models of kidney disease associated with glomerular hyperfiltration.

Key findings have redefined PECs as not merely structural components of Bowman capsule, but as a heterogeneous population with progenitor‐like properties and regenerative potential [4, 66, 67, 68, 69, 70, 71]. This specific subset retaining stem‐like features can be recruited to the glomerular tuft following podocyte injury and acquire phenotypic characteristics of podocytes [68, 72]. However, while these cells support regenerative functions during mild injury, they also contribute to pathogenic crescent formation in severe or chronic conditions [68, 70]. This dynamic ability of PECs to transition from passive structural elements to active regenerative participants underscores their role as regulatory elements within the glomerular microenvironment, modulating pressure‐driven cellular interactions and contributing to the mechanobiological dialogue critical for maintaining filtration barrier integrity and responding to glomerular stress. Recent studies highlight how mechanical stress experienced by podocytes plays a pivotal role in glomerular epithelial disease pathogenesis by regulating the crosstalk between podocytes, PECs and podocyte progenitors among PECs. Specifically, hyperactivation of the universal mechanotransducers and mechanoeffectors YAP1 and TAZ in podocytes triggers proliferation of PECs and contributes to crescentic glomerulonephritis [73, 74]. However, while an expanding list of biochemical mediators involved in PEC physiology and podocyte‐PEC crosstalk is available [60, 68, 69, 70, 75, 76, 77, 78], few studies have explored how mechanical stimuli modulate podocyte progenitor behavior. We cultured human podocyte progenitors on collagen I‐coated hydrogels with variable stiffness to modulate their mechanical environment and found that cell morphology, proliferation, migration, and differentiation toward the podocyte lineage were highly dependent on mechanical substrate stiffness [79]. In particular, the proliferation rate increased with increasing stiffness of the substrate whereas differentiation into podocytes was suboptimal when the cells were cultured on substrates with stiffness deviating from the physiological range [79]. The importance of mechanical stimuli in podocyte progenitor differentiation was further supported by a study performed under simulate microgravity conditions, thereby reducing the mechanical forces acting on cells [80]. Under these conditions, podocyte progenitors exposed to the differentiative agent all‐trans‐retinoic acid failed to acquire the complex, podocyte‐like cytoskeleton and marker expression typical of podocytes. Importantly, treatment with a calcineurin inhibitor, cyclosporine A, rescued differentiation in microgravity conditions by stabilizing the cytoskeleton [80]. Together, these studies demonstrate that mechanical force sensing is essential for progenitor differentiation into podocytes. Considering the changes in mechanical forces that occur in the glomeruli when the capillary tuft expands under pressure, it is plausible that PECs and progenitors engage stretch‐activated pathways to preserve the integrity of both capsule and the filtration barrier. However, the mechanosensory equipment of PECs and podocyte progenitors has been poorly characterized.

Historically, primary cilia have been the first mechanosensory identified in PECs. Indeed, like many other cell types in the body, PECs are equipped with primary cilia—microtubule‐based organelles which function as both chemo‐ and mechano‐sensors [81]. In the kidney, primary cilia protrude from the surface of cells (one per cell) into the lumen, where they detect fluid flow. More recently, it was discovered that unilateral nephrectomy induces elongation of primary cilia not only in tubular epithelial cells but also in parietal cells of the remaining kidney, suggesting that PECs sense changes in urine flow and, like the tubular epithelial cells, may trigger an adaptive response [82, 83].

**FIGURE 5:**
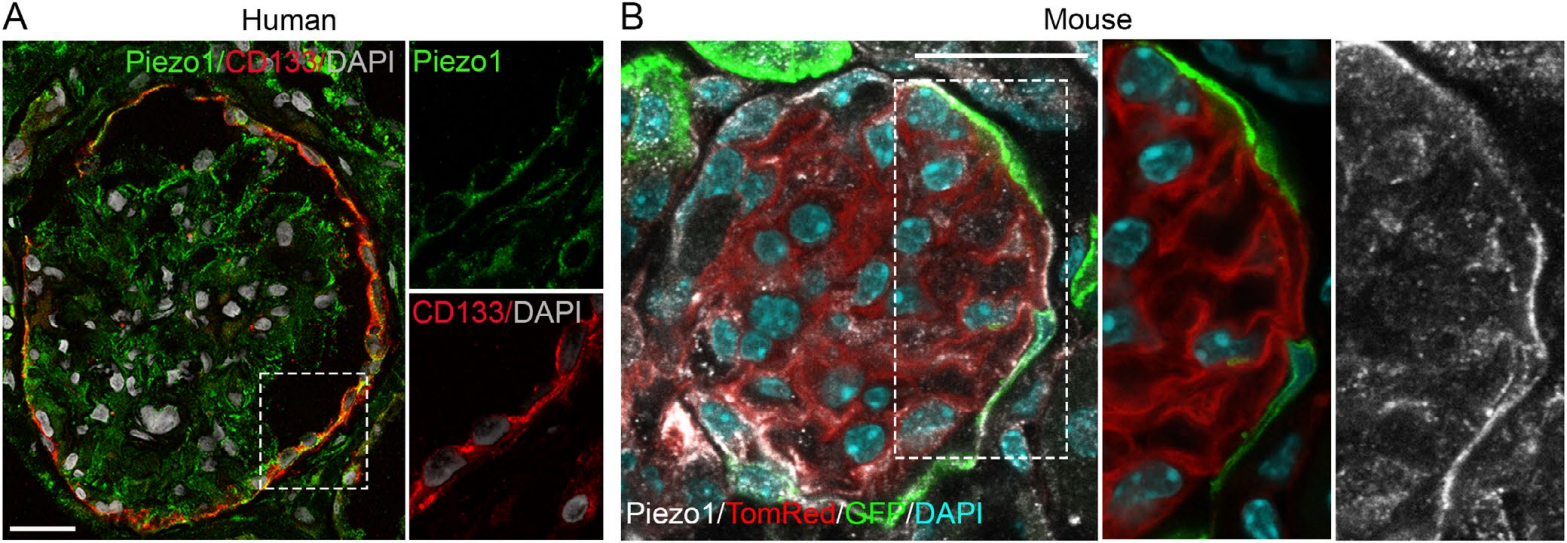
Piezo1 expression in human and mouse podocyte progenitors. (A) Immunofluorescence of a human glomerulus showing Piezo1 (green) co‐localized with the podocyte progenitor marker CD133 (red). (B) Immunofluorescence of a mouse glomerulus showing Piezo1 (white) co‐localized with GFP (green) expressed under the control of podocyte progenitor‐specific promoter Pax2 (modified from Melica et al. JASN 2025 [42]).

Current research has explored the expression of Piezo1 mechanosensing channels in PECs. Immunofluorescence in mouse and human kidneys showed that Piezo1 is detectable in the PECs (Figure 5A,B) and in podocyte progenitors localized among the PECs, identified by their expression of the specific marker CD133 [42]. This finding was further validated by single‐cell RNA sequencing on mouse glomeruli, which revealed variable expression of Piezo1 among PECs and in podocyte progenitors [42]. These data suggest that not only podocytes but also PECs and podocyte progenitors have the capacity to sense stretch, pressure, and other mechanical changes through Piezo1 [42, 43]. However, the mechanotransduction pathways and the cellular responses activated remain largely unexplored. In vitro and in vivo studies on Piezo1 function in podocyte progenitors revealed that modulating Piezo1 expression or activity influences their proliferative and regenerative behavior [42]. In primary cultures of human podocyte progenitors, Piezo1 expression increased in response to shear stress and substrate stiffness. Silencing Piezo1 abolished stiffness‐induced proliferation, indicating Piezo1 as a mechanosensor that regulates podocyte progenitor response [42]. Additionally, Piezo1 silencing orchestrated F‐actin cytoskeleton organization, nuclear shape, and focal adhesion dynamics through RhoA and Rac1 signaling [42]. These findings are in line with the study by Gudipaty et al. [52] who demonstrated that Piezo1 acts as a homeostatic sensor in epithelial cells. In regions of low cell density, Piezo1 promotes cell division in response to stretch, whereas in crowded regions it triggers extrusion and apoptosis. Interestingly, although both processes require Piezo1 at steady state, the type of mechanical force determines the outcome: stretch induces cell division, whereas crowding induces extrusion. This provides a further striking example of Piezo1 activity regulating opposite outcomes based on mechanical context [52].

The role of Piezo1 in podocyte progenitors in vivo has been further evaluated in a podocyte progenitor‐specific Piezo1 KO mouse model (Pax2‐Piezo1‐KO mice). Upon inducing podocyte injury with the ADR model, the KO mice exhibited worsened glomerular damage, increased proteinuria with reduced foot process coverage, a functional phenotype attributable to the inability of Piezo1‐deficient progenitors to generate fully functional podocytes [42]. Indeed, while in WT mice the Pax2 cells generated cells inside the tuft with recognizable central body, nucleus, and cytoplasmic processes resembling podocytes, in the Pax2‐Piezo1‐KO mice we observed the presence of fragmented structures reminiscent of cell debris [42]. The finding of recruitment of PEC‐derived cells to the glomeruli in the hypertensive podocyte‐Piezo1 KO mice underlines the necessity to explore the role of Piezo1 in PECs and in podocyte progenitors in different pathologic conditions [46].

In this framework, PECs may be compared to an impermeable aquiclude: by lining Bowman capsule, they define the boundary that constrains filtrate movement and separates the high‐pressure glomerular space from the surrounding tissue. This analogy is useful because it emphasizes how PECs, much like aquicludes in hydrogeology, provide structural containment and influence local pressure dynamics, thereby contributing to the regulation of ultrafiltration and the maintenance of glomerular homeostasis.

## 5 Conclusions

Understanding the glomerulus through the lens of hydrogeology provides a compelling paradigm to study its biomechanical complexity. Like an aquifer system bounded by aquicludes and monitored by piezometers, the glomerulus integrates mechanical barriers and mechanosensors to regulate flow, maintain integrity, and adapt to stress. Piezo1 and related channels emerge as pivotal elements in this system—transducing physical forces into biological responses that determine podocyte and progenitor fate. Future studies unraveling this mechanosensory network may open novel avenues for diagnosis and therapy in glomerular disease, grounded in the biomechanics of pressure, flow, and force.

## Ethics Statement

The authors have nothing to report.

## Conflicts of Interest

The authors declare no conflicts of interest.

## Data Availability

Data sharing not applicable to this article as no datasets were generated or analyzed during the current study.
